# Fate and transport of radioactive gypsum stack water entering the Floridan aquifer due to a sinkhole collapse

**DOI:** 10.1038/s41598-018-29541-0

**Published:** 2018-07-30

**Authors:** Daljit Sandhu, Arvind Singh, Steven J. Duranceau, Boo Hyun Nam, Talea Mayo, Dingbao Wang

**Affiliations:** Department of Civil, Environmental, and Construction Engineering, University of Central Florida, 12800 Pegasus Dr. Suite 211, Orlando, FL 32816-2450 USA

## Abstract

Groundwater aquifers are an essential source of drinking water, and must be protected against contamination. Phosphogypsum stacks originating from the processing of phosphate rock contain small amounts of radionuclides, such as ^226^Ra. In September 2016, a sinkhole located beneath a phosphogypsum stack collapsed under central Florida’s carbonate karst terrain, where the aquifer is mostly confined, raising concern over water quality in the regions nearby. Monitoring and modeling the transport of the contaminated plume is vital to ensure drinking water criteria are met and to improve decision making regarding treatment. To achieve this, a geochemical modeling using PHREEQC software was employed to investigate the trajectory of the plume based on hydraulic and hydrologic conditions. Adsorption was simulated as a removal mechanism that could further reduce the intensity of the plume. The aquifer’s response to the release of contaminated water from the collapsed stack was quantified by simulating a number of scenarios, including variable radionuclide leakage quantities. Results suggest that it may take between 11–17 years and between 5.2 to 8.3 km from the sinkhole leak to reduce radionuclide concentrations to previous levels. Coupling the adsorption effect by minerals in Floridan aquifer (e.g. ferrihydrite, carbonate) can reduce radionuclide migration time to 9–16 years and distances between 4.3 to 7.8 km from the sinkhole leak. It can also reduce the distance needed to lower radionuclide concentrations, though not significantly. Additionally, due to the complexities of soil chemistry, the importance of groundwater remediation is emphasized.

## Introduction

**Figure 1:**
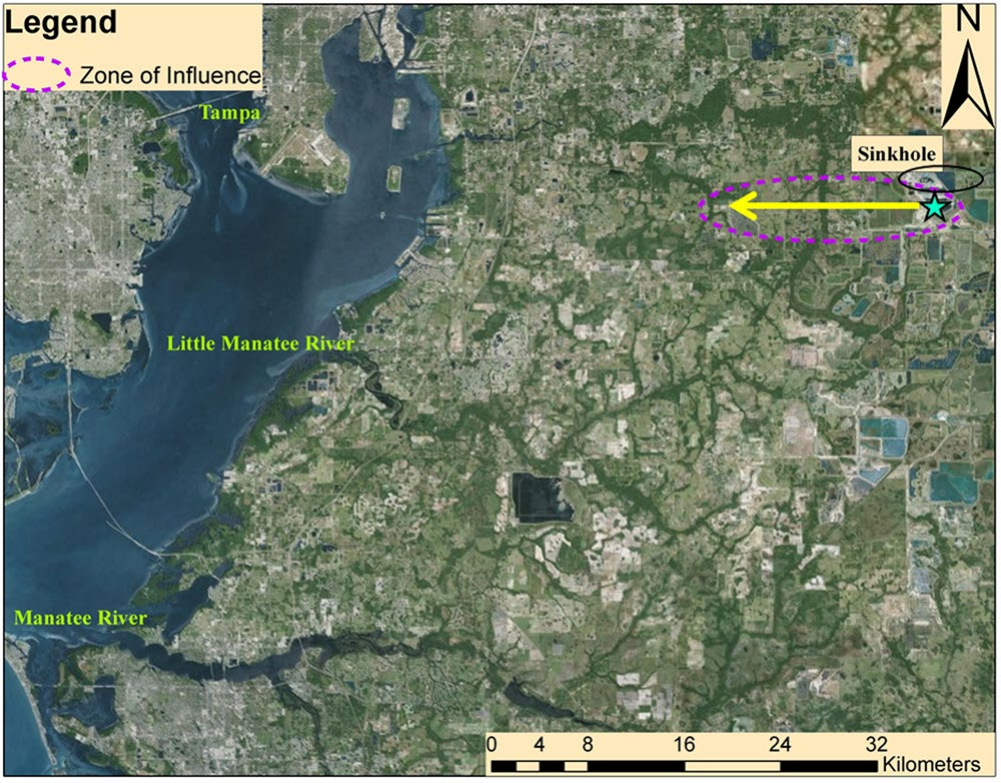
Location of the sinkhole as depicted by the blue star, and the general direction of groundwater flow shown by the yellow arrow. The area circled in black represent approximate well locations. The dashed area in purple is a conceptual representation of the zone of influence of the plume after 20 years, based on advection, diffusion, and adsorption. The zone of influence would be smaller if any wells in the region are active. (Figure created using ESRI ArcGIS 10.0 software, https://www.esri.com/en-us/arcgis/about-arcgis/overview. World Imagery basemap is included in ArcGIS 10.0 and is attributed to ESRI, DigitalGlobe, GeoEye, Earthstar Geographics, CNES/Airbus DS, USDA, USGS, AEX, Getmapping, Aerogrid, IGN, IGP, swisstopo, and the GIS User Community).

The presence of phosphogypsum stacks can be harmful to natural aquifers, potentially contaminating and degrading groundwater below that may serve as a source of drinking water. The gypsum in phosphogypsum stacks is created as a by-product after phosphorous acid is obtained from phosphate rock and processed in industrial facilities, and these stacks contain radionuclide amounts, such as radium^1^. Central Florida is home to numerous gypsum stacks and is also prone to sinkholes due to karst geology^2^. In September 2016, a sinkhole spanning 13.7 m (45 ft) in diameter damaged the liner system at the base of a phosphogypsum stack, causing an opening that allowed an estimated 813,000 cubic meters (215 million gallons) of waste fluids to leak into the Floridan aquifer (location shown in Fig. 1), immediately raising concerns over the extent of radionuclide contamination to the area’s drinking water supply.

The Mosaic Company, owner of a phosphate fertilizer facility near Mulberry, Florida, acted quickly to prevent the migration of the by-products that leaked into the sinkhole. The Florida Department of Environmental Protection (FDEP) and the U.S. Environmental Protection Agency (USEPA) defines the maximum contaminant level (MCL) of combined radium (^226^Ra and ^228^Ra) as 0.185 Bq/L for drinking water. There are naturally occurring amounts of radium in soils which vary according to the location and the configuration of the terrain. Observed radioactive quantities in the nation’s aquifers vary from 0.037 to 0.185 Bq/L (1–5 pCi/L)^3^. In general, gypsum stacks are acidic, and have a pH ranging from 1.5 to 2.0. They also contain high concentrations of fluoride, sulfate, phosphate, and sodium^2^. A sinkhole event beneath a gypsum stack may cause radionuclides to leak and migrate in the aquifer. Therefore, it is necessary to monitor and sample the total mobilized radium contamination in aquifers potentially affected by this incident to ensure that requirements for safe drinking water are met.

In 1994, a prior sinkhole occurred under a phosphogypsum stack in proximity of the more recent event. Similarly, preventing contamination of the Floridan aquifer was a high priority. To achieve this, angle drilling and injection of cement grouting at the throat of the sinkhole just ahead of the aquifer were performed to prevent further leakage. In addition, surrounding recovery wells were used to extract and dispose contaminated water flowing in the aquifer. Fuleihan *et al*.^2^ argued that the contaminated water was contained on site and pre-sinkhole conditions were restored through this process (with an estimated cost of $6.8 million, which translates to $10.5 million today), however they also noted that the production wells might require several additional years to remove the contaminants from the aquifer^2^.

It has been suggested that the geology and the associated distributions of minerals for Florida in particular can enhance the understanding of the behavior of radioactive contaminants flowing within aquifers^4,5^. For example, understanding the distribution of minerals is a key for predicting the transport capabilities of radionuclides, in this case, radium because those minerals lead to geochemical reactions including adsorption, precipitation and complexation, which affect its advective and diffusive transport. It is noted that the adsorption process is speculated to govern radium dissolution at small concentrations^4^. This can be simulated by modeling the reactions and transport capabilities between radionuclides and minerals. The minerology has been investigated in southwest central Florida, which is in the general vicinity of the study area. According to X-ray diffractions (XRD) from core samples, it is mostly comprised of calcite and dolomite, with some clay minerals in between^5^.

Various models have been developed to quantify the geochemistry and transport of elements in groundwater systems. Some examples are WATEQ4F, pH-REdox-EQuilibrium (PHREEQC) (from the United States Geological Survey, USGS)^6^, the Geochemist’s Workbench, and Metal Speciation Equilibrium for Surface and Ground Water Model (MINTEQA2) (from USEPA). The PHREEQC model is of particular interest for this study because of its common use and extensive transport modeling capabilities. For modeling surface complexation studies, PHREEQC incorporates data from Dzombak and Morel^7^, where sorption reactions can be modeled using the mineral hydrous ferric oxide^7,8^. The PHREEQC transport feature is governed by one-dimensional advection-diffusion equation^9,10^. Previous studies have utilized the PHREEQC model to understand the behavior of radionuclides in coastal Brazil^11,12^, geochemistry in Mexico^13^, and radionuclide transport in Germany^14^. For example, Navarro and Carbonell^15^ investigated aquifer contamination due to waste dumping by using PHREEQC to simulate reactions, speciation, and transport in Spain^15^. PHREEQC was also used to investigate the geochemical controls that led to radium activity levels in excess of the 0.185 Bq/L USEPA limit in Wisconsin. From this study, it was determined that sorption reactions that are assumed to control radium can also control barite^16^, the precipitation of multicomponent solids can also have an impact on contaminant remediation^16^, though not studied here. Zhu *et al*.^17^ constructed a transport model using PHREEQC to study groundwater plume attenuation in a pond. In essence, PHREEQC has the capability to model water and contaminant transport processes and establish a connection with real world physical hydrogeochemistry^18^. In some cases, during the drinking water monitoring process, it may be necessary to consider a sinkhole collapse event beneath radioactive gypsum stacks and the resulting transport of leaked radium in the aquifer due to the effects of both advection-diffusion and adsorption, as advection-diffusion alone may not always be enough to explain dilution of radium concentrations. In this study we use the PHREEQC model to investigate the transport of radioactive material through the groundwater aquifer system in central Florida.

## Results and Discussion

**Figure 2:**
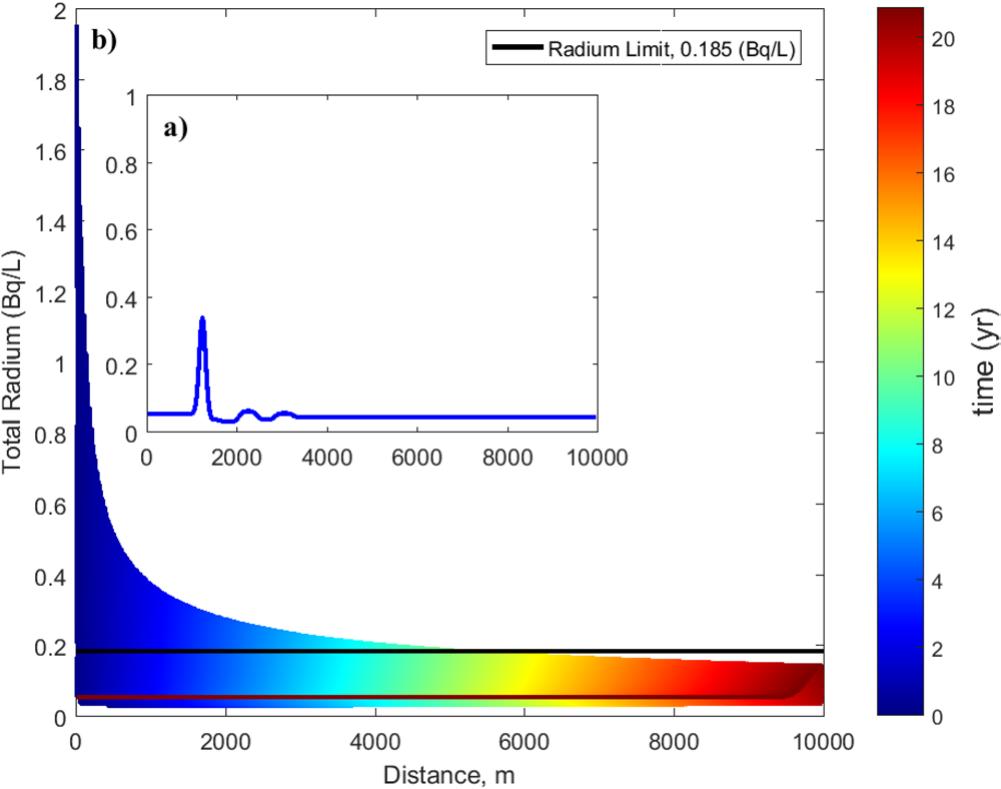
Sample output graph of radium concentration vs. distance, considering advection and diffusion for (**a**) a time instant and (**b**) the complete simulation time. Cooler colors and warmer colors represent breakthrough curves near the initial and final simulation times, respectively. The black line shows the radium concentration limit whereas the lower dark red curve shows the plume concentration at the end of the simulation. The constant dark red portion represents background conditions.

PHREEQC was used to model one-dimensional radium transport across a range of distances and conditions. PHREEQC internally computes the velocity of a plume as the cell length divided by the time step. This velocity (1.31 m/day) for 1 unit hydraulic gradient is estimated based on the reported hydraulic conductivity (~392 m/day) and porosity (0.20)^19^. This is utilized to mimic the contaminant plume being transported along the hydraulic gradient. A well for limited public use is situated at just over 4 km west from the sinkhole, according to the FDEP. Thus, 4 km was chosen as the length of the cell domain as an initial conservative, worst-case scenario to determine whether the radioactive plume will hit the nearest active well. If concentration levels are too high, the model would be extended to see at what distances the concentrations will be adequately reduced. This distance is referred to as the “safe distance”. Plotting results in Bq/L allows one to compare the radium transport concentration with the USEPA drinking water limit of 0.185 Bq/L ($\approx 5\ast {10}^{-9}$ mg/L) and then assess whether the radium is below this threshold. A sample output plot of radium transport vs. distance with the breakthrough curves is shown in Fig. 2. The inset in Fig. 2 shows a sample plot of a breakthrough curve at an instant in the simulation. For each scenario, the maximum value of the modeled breakthrough curves at each cell is plotted separately as an upper envelope. Figure 2 presents a case for advection and diffusion at distances less than ~5.2 km, hence the content is above the threshold concentration (Fig. 2). Distances greater than ~5.2 km indicate that the quantities are below the threshold concentration.

According to the FDEP, the radium activity at the gypsum stack under normal condition is 79 pCi/L (2.93 Bq/L)^20^; thus, the radioactive transport of radium was modeled using this value with and without adsorption. Then, as a severe case, in an attempt to represent the sinkhole collapse, a value of 100 pCi/L (3.70 Bq/L) of radium was simulated with and without adsorption to study the differences in the plume transport.

### Ferrihydrite cases

**Figure 3:**
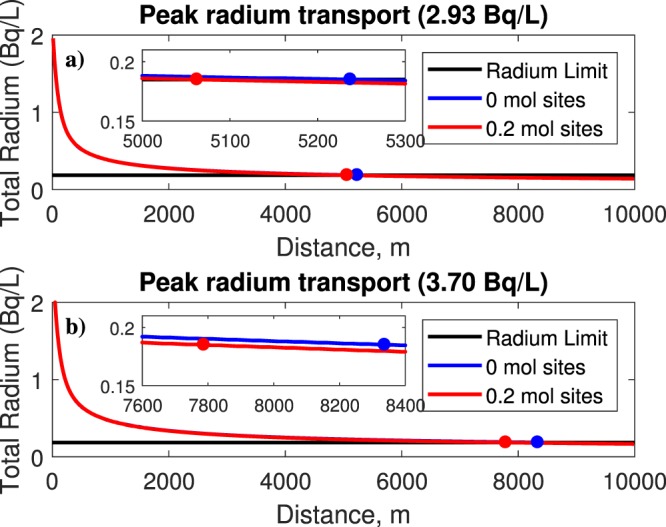
Output plots of radium transport with weak surface ferrihydrite at leaks of (**a**) 2.93 Bq/L and (**b**) 3.70 Bq/L. As expected, a longer distance is needed to dilute the concentration of 3.70 Bq/L. In both cases, accounting for adsorption reduces the distance needed for dilution.

**Figure 4:**
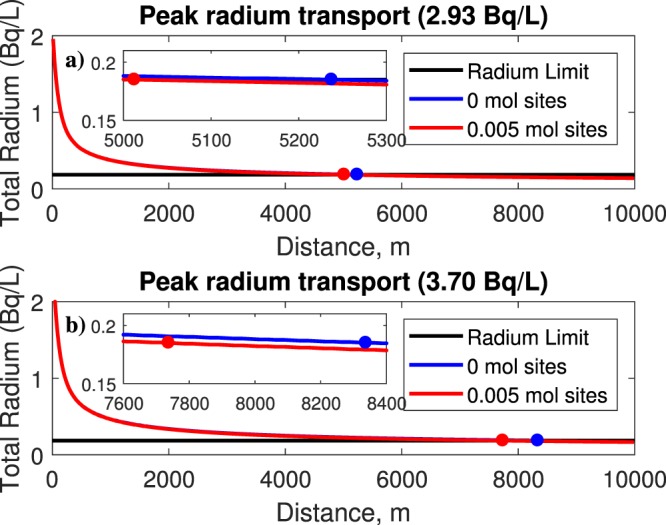
Output plots of radium transport with strong surface ferrihydrite for leaks of (**a**) 2.93 Bq/L and (**b**) 3.70 Bq/L. Similar to the previous plot, adsorption (red dots) slightly reduces radium concentrations.

In this section, we explore the radium transport with ferrihydrite. Ferrihydrite can be represented as a weak or a strong binding surface type. For this case, adsorption is simulated using ferrihydrite only, and is modeled with the well data. The simulation with 0 mol sites represents advection and diffusion only. Using a value greater than 0 mol sites will enable the adsorption effect. Here, a value of 0.2 mol sites was used for weak ferrihydrite, based on the data from Dzombak and Morel^7^. Figure 3 shows the behavior of radium transport reacting with weak surface binding ferrihydrite using leaks of 2.93 Bq/L and 3.70 Bq/L. In this case, the ferrihydrite reacting without adsorption requires a longer distance to reduce radium concentrations for both the 2.93 and 3.70 Bq/L scenarios; whereas with adsorption, these distances are reduced. For example from Fig. 3a it can be observed that the safe distances after a 2.93 Bq/L leak are 5.24 km and 5.06 km without and with adsorption, respectively. The contaminant plume would reach those distances at times of 11 and 10.6 years, respectively. For the case of 3.70 Bq/L, the safe distances without and with adsorption are 8.35 km and 7.79 km, respectively (Fig. 3b). The associated times for the plume to reach those distances are 17.4 and 16.3 years (see Table 1). Figure 4 shows the radium behavior with strong surface binding ferrihydrite, again for leaks of 2.93 Bq/L and 3.70 Bq/L. To simulate adsorption for the strong surface ferrihydrite, 0.005 mol sites are used for the surface, again based on data from Dzombak and Morel^7^. Although not easily seen, the strong binding ferrihydrite performed slightly better in terms of reducing concentrations. However, the influence of ferrihydrite on contaminant binding is minor. Figure 4a indicates safe distances without and with adsorption of 5.24 km and 5.01 km, after just 11 and 10.5 yrs, respectively, for the case of 2.93 Bq/L, whereas for 3.70 Bq/L (Fig. 4b) the safe distances without and with adsorption are 8.35 km and 7.74 km, after 17.4 and 16.2 yrs, respectively. These results suggest that the strong surface binding ferrihydrite is capable of greater adsorption. Previous studies also suggest that other constituents in the solution may compete with each other to bond with the mineral, which could minimize its impact^11,21^. From other studies, a low pH in the solution may hinder adsorption capacity^11^. A summary of these values is listed in Table 1.

**Table 1:** List of distances (km) and times (yrs) in which radium concentrations would meet drinking water criteria for all simulations shown.

Bq/L	mol sites	Distance, km	Time, yrs
**Weak Ferrihydrite**			
2.93	0	5.24	11
	0.2	5.06	10.6
3.70	0	8.34	17.4
	0.2	7.79	16.3
**Strong Ferrihydrite**			
2.93	0	5.24	11
	0.005	5.01	10.5
3.70	0	8.34	17.4
	0.005	7.74	16.2
**Carbonate (5 sites/nm** ^**2**^ **)**			
**Bq/L**	**Specific Surface Area (m** ^**2**^ **/g)**	**Distance, km**	**Time, yrs**
2.93	0	5.24	11
	10	4.96	10.3
	22	4.36	9.1
3.70	0	8.34	17.4
	10	7.79	16.3
	22	6.51	13.6

### Carbonate case

**Figure 5:**
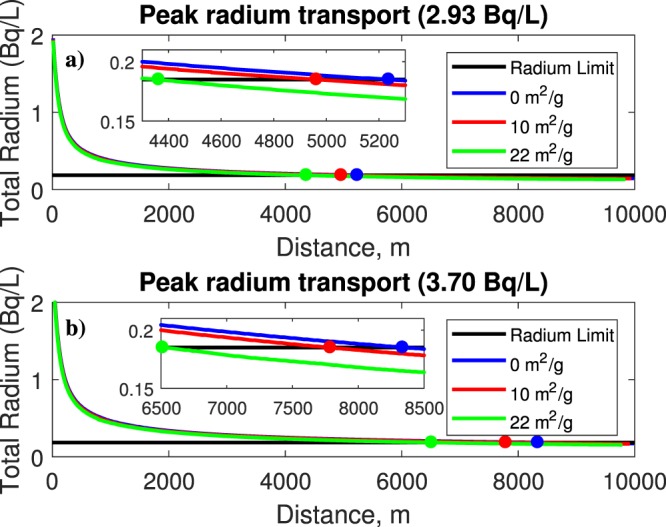
Output plots of radium transport with carbonate using leaks of (**a**) 2.93 Bq/L and (**b**) 3.70 Bq/L. In general, the higher the specific surface area is, the faster (i.e., shorter distance) the dilution of the plume.

Figure 5 shows the radium transport with carbonate for the case of 2.93 Bq/L and 3.70 Bq/L as a function of distance. For these scenarios, a site density of 5 sites/nm^2^ was used, which has been used previously according to the Rossendorf Expert System for Surface and Sorption Thermodynamics (RES^3^T) database. Two scenarios of specific surface area of calcite are used, i.e., 10 m^2^/g and 22 m^2^/g, also obtained from the RES^3^T database. This range of known surface areas from the RES^3^T database was assumed to apply to the study area due to the presence of carbonate rock in Florida. As can be seen from Fig. 5a, the safe distance without considering adsorption is about 5.24 km, which corresponds to about 11 yrs. Adsorption with a specific surface area of 10 m^2^/g reduced the safe distance to 4.96 km, which would take the plume about 10.3 yrs to reach. As expected, the higher the specific surface area, the higher the adsorption, which would imply that larger carbonate minerals (i.e., specific surface area) would have the potential to reduce the contaminant plume in the central Florida area via adsorption. Increasing the surface area to 22 m^2^/g further reduced the safe distance to 4.36 km, after 9.1 yrs. Similarly, this effect can be seen for the 3.70 Bq/L scenario, in Fig. 5b. Without adsorption, the safe distance would be 8.35 km, or 17.4 yrs. At 10 m^2^/g surface area, the safe distance is reduced to 7.79 km, 16.3 yrs, and at 22 m^2^/g, the safe distance is further reduced to 6.51 km, or 13.6 yrs. These values are also listed in Table 1. Due to the frequent presence of calcite (i.e. limestone) in the Floridan aquifer, it appears that the calcite would be capable of resisting the flow of contamination, but the time scale is too large to observe this. If there was an abundant mass amount of mineral, perhaps that would further reduce concentration levels, but that would not significantly reduce the risk associated with this event, regardless of how much radium leaked into the aquifer.

Overall, for the observed cases, the results suggest that there is a risk of drinking water contamination due to radionuclide transport. Although both the weak and strong binding ferrihydrite mineral performed similarly, the result can change depending on the competitive nature of the constituents in the soil, which can be seen in past analyses^22^. A previous study showed that radium levels in Tampa Bay are low. Our analysis would appear to follow the same trend^23^. The carbonate performed better than the ferrihydrite minerals, but since the time scale is long, i.e. on the order of tens of years, and due to drinking water processing regulations, additional external resources should be leveraged to safely contain the contaminated waste. This further reinforces the importance of safely containing phosphogypsum stack wastes that may threaten natural resources, such as drinking water.

## Summary and Conclusions

In this study we simulated the transport of radioactive contaminant waste in the Floridan aquifer. To model the reduction of the concentration of radium, advection, diffusion, and adsorption were simulated using the PHREEQC transport model. Based on the hydrogeological nature of Florida, our results suggest that it would take between 11–17 years and about 5.2 km to 8.4 km from the sinkhole leak to naturally reduce concentrations at or below the drinking water threshold of 0.185 Bq/L. Including the adsorption effect using entities like ferrihydrite (with either weak or strong binding surfaces) and carbonate may help to moderately reduce the time needed to lower concentrations, i.e. to 9–16 years. The safe distances associated with these times range from 4.4 km to 7.8 km.

Although ferrihydrite is commonly occurring in nature, it is likely not as frequent as calcite in central Florida. Despite that, ferrihydrite was simulated based on the available data by Dzombak and Morel^7^ and Sajih *et al*.^21^. Based on the simulations, ferrihydrite can treat the contaminant waste, however a long time period is needed to ensure that radium concentrations reach levels below the threshold determined by the USEPA. For weak ferrihydrite minerals and a 2.93 Bq/L leak, it would take a distance of more than 5 km and about 10–11 years for the concentration to be attenuated. A leak of 3.70 Bq/L would require distances from 7.8 to 8.4 km and 16–17 years for remediation. For strong ferrihydrite minerals, results were slightly different. For a 2.93 Bq/L leak, it would take over 5 km and under 11 years to remediate the plume. For the 3.70 Bq/L case, 7.4 to 8.4 km and 16-17 years would be necessary to attenuate the waste. For the carbonate case, distances from 4.4 to 5.2 km and 9–11 years are sufficient for a 2.93 Bq/L scenario, and distances from 6.5 to 8.4 km and 13–17 years would be sufficient for a 3.70 Bq/L leak. From the observations, it appears that calcite may be the best mineral to counteract the radioactive plume. Although calcite may be commonly occurring in the study area, additional efforts (i.e., barriers or cement injection) may be needed to contain and treat the contaminant waste to meet the drinking water criterion (0.185 Bq/L) before potentially hitting a pumping well. The amount of abundant minerals in the aquifer can affect the overall remediation process; however it is complex to quantify the amount of potential adsorbents in the soil. From previous studies, a low pH and the presence of other elements and compounds in the solution may induce competition among them, which may then in turn affect the potential of adsorption occurring in the soils^21,22^.

Based on the cases presented, there is uncertainty in the fate of the contaminant waste after the sinkhole collapse. Several factors, hydrological and chemical, play roles on influencing how radionuclides are transported. The large-scale nature of this event alone poses considerable risk. Perhaps if the sinkhole event was small-scale, the outlook may have been favorable. Due to difficulties and complexities in knowing the exact conditions in the aquifer, a range of possibilities must be analyzed to aid in developing potential solutions. This stresses the importance of safely containing contaminant waste before natural resources, such as drinking water, are harmed. It is probable that carbonate, calcite, and ferrihydrite are present and significant to adsorption processes; several of these processes are simulated to increase the likelihood that the actual behavior of the plume transport falls within the cases.

While characterizing aquifer and chemical conditions may be complex, it is essential to providing a basic understanding of contaminant transport following natural hazardous events through observation and simulation. Thus, this is a necessary step in identifying potential threats imposed on surrounding systems, and ultimately improving best management practices.

## Methodology

Radium transport is modeled by considering the effects of advection, diffusion, and adsorption using the PHREEQC model. In general, PHREEQC simulates one-dimensional transport of contaminants by accounting for the element concentrations, which can be measured from well samples. In addition, PHREEQC defines surface reactions that can mimic the adsorption effect. In this study, several simulations are considered based on various possible ways adsorption can occur in the Floridan aquifer, and the concentration of radionuclides is monitored near the sinkhole site. Table 2 lists the chemical reactions used for adsorption simulation, these reactions are explained in more detail in this section. Specifically, the concentration of radium is modelled to determine whether it exceeds the USEPA MCL. This is accomplished by assessing how the radium concentration changes due to the effects of advection, diffusion, and adsorption.

**Table 2:** Chemical reactions simulating surface adsorption, where ‘≡’ denotes a surface.

Mineral case	Reaction	Log K
Carbonate	Ra^+2^ + CO_3_^−2^ = RaCO_3_	2.50
Ferrihydrite (weak surfaces)	≡OH + Ra^+2^ = ≡ORa^+^ + H^+^	−5.67
Ferrihydrite (strong surfaces)	≡OH + Ra^+2^ = ≡OHRa^+2^	6.66

### Study area description

The sinkhole occurred in Mulberry, Florida, approximately 30 miles outside Tampa. According to Tampa Bay Times^24^, it was estimated that the sinkhole was 45 ft (~14 m) wide and 300 ft (~91 m) deep. The sinkhole caused roughly 215 million gallons (900 million L) of gypsum to be dumped into the upper Floridan aquifer, the major source of drinking water for the state.

The Floridan aquifer is mostly confined in central Florida^25^, and consists of carbonate karst terrain, which is prone to sinkholes. At roughly 300 ft (~91 m) below ground level, the aquifer is comprised of Suwannee and Ocala limestone^26^. The soil within the property is primarily made up of fine sand and also clay, though to a lesser extent. However, from this soil configuration, the carbonate minerals are relevant for this study. Florida has a fairly flat topography, however as observed from the study site, groundwater tends to flow in the west direction, towards Tampa Bay (see Fig. 1).

In the general case for adsorption, the ferrihydrite mineral can serve to adsorb pollutants in natural systems^27^. In addition to advection and diffusion, this can further help reduce concentrations of radium in aquifers. Furthermore, from previous studies, iron oxyhydroxides are other common adsorbents found in nature due to their high surface areas and sorption capacities^27,28^. However, in the southwest Florida vicinity, carbonate minerals, such as calcite and dolomite, are quite common, and can be seen in the X-ray diffractogram^5^. Consequently, reactions of radium with ferrihydrite, using available data, and carbonate were relied on to simulate adsorption in this study.

### Model description and data collection

PHREEQC simulates advection and diffusion as follows:

$$\frac{\partial C}{\partial t}=-\,v\frac{\partial C}{\partial x}\,+{D}_{L}\frac{{\partial }^{2}C}{\partial {x}^{2}}-\frac{\partial q}{\partial t}$$

where *C* is concentration in water, *t* is time, *v* is pore water flow velocity, *x* is the distance, *q* is the concentration of the solid phase, and *D*_*L*_ refers to the hydrodynamic dispersion coefficient (${D}_{L}={D}_{e}+{\alpha }_{L}v$), where *D*_*e*_ is the diffusion coefficient, *v* is the pore water flow velocity, and *α*_*L*_ is the dispersivity^6^.

**Figure 6:**
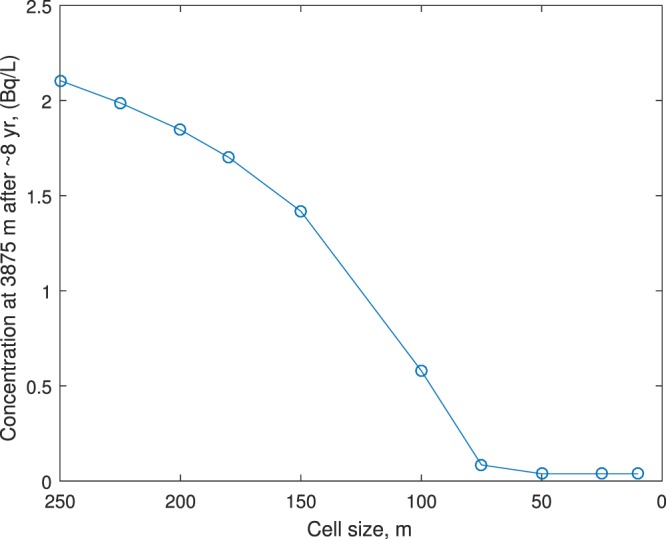
Sensitivity of radionuclide concentration to cell size. The initial concentration used was 2.93 Bq/L.

In the PHREEQC model, the contaminant source is modeled as an instantaneous leak into the aquifer through the sinkhole, followed by background groundwater flushing the contaminant plume along the flow path. The exact amount of radionuclide waste that leaked into the aquifer is unknown, but the normal stack condition concentration as reported by Mosaic to the FDEP^20^ was 2.93 Bq/L as combined radium. To simulate a worst-case scenario, 3.70 Bq/L of leakage is simulated. These values are used as the input concentrations at the sinkhole. The model structure itself consists of a one-dimensional (1D) grid, discretized into a number of cells. Each cell has a user defined length and time step. Before deciding on an adequate number of cells, a sensitivity analysis was performed to quantify the effect of different cell sizes on model output, under the same chemical compositions. Figure 6 shows the concentration output from the model as a function of cell size. As can be seen from Fig. 6, the output concentration is dependent on cell size when cell size is greater than 75 m, but is not sensitive to cell size when cell size is reduced to 50 m. Therefore, a cell size of 25 m is used in this study. The corresponding time step for a 25 m cell size is 1,644,737 sec, or roughly 19 days. This value is important because should the plume approach the nearest well, the well may need to be shut down. Also, the Little Manatee and Manatee Rivers are positioned in the path of the groundwater flow, therefore it is important to know if and when the contaminant plume would hit the rivers.

Next, the chemical concentrations and properties of well samples were provided as inputs to the model. In addition, existing data of constituents from active wells surrounding the site analyzed by FDEP as well as additional well samples were used in the PHREEQC model. To be consistent across all data, radium concentration (pCi/L) was converted into mg/L. Table 3 shows the solution composition and properties at the sinkhole. As the leak was mostly acidic water, a pH value of 2 was used for the gypsum stack, which was near the value reported by FDEP^20^. In addition, the density of the phosphogypsum leak was assumed to be 2.45 g/cm^3^, as the waste was heavily concentrated. This value was near the range reported in SENES^29^.

**Table 3:** Conditions and solution composition at sinkhole.

Constituent	Units	Value
pH		2
Temperature	C	22
Density	g/cm^3^	2.45
Aluminum	mg/L	241
Calcium	mg/L	1962
Chloride	mg/L	190
Fluoride	mg/L	13207
Iron	mg/L	233
Magnesium	mg/L	616
Manganese	mg/L	11.1
Ammonia Nitrogen	mg/L	872
Potassium	mg/L	295
Phosphorous	mg/L	9207
Radium	Bq/L	2.93, 3.70
Sodium	mg/L	2109
Sulfate	mg/L	8024

Well data for the background and flow path conditions were collected for the transport simulations. The sampled wells are operated by Polk County Utilities and Tampa Bay Water to analyze the water quality post-spill. Data collection was carried out in agreement with Standard Methods (2017), Table 4 shows the well data in the vicinity of the sinkhole^30,31^. The pH, temperature, ^226^Ra, sulfate, fluoride, barium, calcium, and sodium descriptions were used as inputs to PHREEQC. In the aquifer, the groundwater density was assumed to be 1.02 g/cm^3^, a conservative estimate^32^. Upon mixing, the plume density in the aquifer was assumed to be 1.75 g/cm^3^. For the simulations, different well data were applied throughout the model. The cell dispersivity was assumed to be 2 m, due to the low hydraulic gradient and based on the data by Gelhar^33^, and the diffusion coefficient representative of the study area was assumed to be $9.9\ast {10}^{-11}$ m^2^/s^34^. Simulations were run with the llnl.dat database, a file that contains specific thermodynamic data^10^.

**Table 4:** Solution composition for background conditions (in which maximum values are used), and conditions in close proximity to the flow path. A well density of 1.02 g/cm^3^ is used for the model simulations.

Sample Description	Unit	Background	Well 16	Well 17	SCHM 5D	SCH17	SCH16
Conductivity	uS					526	411
**pH**		7.65	7.5	7.56	7.59	7.56	7.59
**Temp**	C	26.1	24.2	27.3	25.9	27.3	25.9
Turbidity						0.4	0.41
H2S						2.50	2.10
Gross alpha	Bq/L (pCi/L)		0.08 (2.2)	0.21 (5.7)	0.06 (1.6U)	0.10 (2.60)	0.13 (3.60)
**Ra-226**	Bq/L (pCi/L)	0.02 (0.6)	0.02 (0.5)	0.04 (1)	0.02 (0.6)	0.03 (0.90)	0.03 (0.70)
Ra-228	Bq/L (pCi/L)		0.03 (0.8U)	0.03 (0.8U)	0.03 (0.8U)	0.04 (1.10)	U
Uranium	Bq/L (pCi/L)		0.01 (0.4U)	0.02 (0.6U)	0.02 (0.5U)	U	U
**Sulfate**	mg/L	58.2	13.6	66.2	70.6	69.5	11.9
**Fluoride**	mg/L	0.29	0.14	0.18	0.24	0.38	0.33
TDS	mg/L		181	260	259	254	174
**Barium**	mg/L	0.01	0.017	0.023	0.012	0.025	0.022
**Calcium**	mg/L	54.1	39.3	54.5	54.5	53.7	40.3
**Sodium**	mg/L	7.01	7.4	7.71	6.88	9.07	8.67

Langmuir and Reese^35^ provided thermodynamic properties and reaction parameters of radium, which can be input into the PHREEQC model. In addition, adsorption reactions of radium onto ferrihydrite for both weak and strong binding surfaces along with their equilibrium constants (log K) have been defined, which are used in this study^21^. Table 2 summarizes the chemical reactions used for adsorption simulation. The values from Dzombak and Morel^7^ and the reactions derived from Sajih *et al*. were applied to the study area. Here, the specific surface area of hydrous ferric oxide was assumed to be 600 m^2^/g^7^. The site densities of weak and strong binding sites were assumed to be 0.2 and 0.005 sites per mol Fe, respectively based on the same database^7^.

Furthermore, since the study area is mostly calcite, the reaction of radium with carbonate was also considered as this would occur in alkaline aquifers. The reaction and equilibrium constant were based on the work of Langmuir and Reese^35^. The approximate specific surface area of carbonate ranges from ~2–22 m^2^/g, according to the RES^3^T database. In general, site density values may range from 1–20 sites/nm^2^ ^36^.

### Data Information

The data used in this study can be requested from authors.
